# Mosquito Salivary Antigens and Their Relationship to Dengue and *P. vivax* Malaria

**DOI:** 10.3390/pathogens13010052

**Published:** 2024-01-05

**Authors:** McKenna M. Howell, Olayinka M. Olajiga, Jenny C. Cardenas, Claudia A. Parada-Higuera, Maria U. Gonzales-Pabon, Lady Y. Gutierrez-Silva, Lucy Jaimes-Villamizar, Brett M. Werner, Jeffrey G. Shaffer, Jennifer A. Manuzak, Berlin Londono-Renteria

**Affiliations:** 1 Arbovirology Laboratory, Department of Tropical Medicine and Infectious Disease, Tulane University, New Orleans, LA 70112, USA; mhowell4@tulane.edu (M.M.H.); carocardenasg@hotmail.com (J.C.C.); 2 Hospital Regional Norte, Tibu, Norte de Santander 547079, Colombia; clau_099@hotmail.com; 3 Hospital Universitario Erasmo Meoz, Cucuta, Norte de Santander 547079, Colombia; maubago94@gmail.com; 4 Hospital Emiro Quintero Cañizares, Ocana, Norte de Santander 547079, Colombia; yuvey716lagusi1982@hotmail.com; 5 Hospital Jorge Cristo Sahium, Villa del Rosario, Norte de Santander 547079, Colombia; lucyjv2009@hotmail.com; 6 College of Science and Technology, Bellevue University, Bellevue, NE 68005, USA; brwerner@bellevue.edu; 7 Department of Biostatistics and Data Science, Tulane University School of Public Health and Tropical Medicine, New Orleans, LA 70112, USA; jshaffer@tulane.edu; 8 Division of Immunology, Tulane National Primate Research Center, Covington, LA 70433, USA; jmanuzak@tulane.edu

**Keywords:** *Aedes*, dengue, *Anopheles*, malaria, mosquito saliva, biomarkers

## Abstract

In tropical areas, the simultaneous transmission of multiple vector-borne diseases is common due to ecological factors shared by arthropod vectors. Malaria and dengue virus, transmitted by *Anopheles* and *Aedes* mosquitoes, respectively, are among the top vector-borne diseases that cause significant morbidity and mortality in endemic areas. Notably, tropical areas often have suitable conditions for the co-existence of these mosquito species, highlighting the importance of identifying markers that accurately indicate the risk of acquiring each specific disease entity. *Aedes* are daytime-biting mosquitoes, while *Anopheles* preferentially bite during the night. These biting patterns raise the possibility of concurrent exposure to bites from both species. This is important because mosquito saliva, deposited in the skin during blood feeding, induces immune responses that modulate pathogen establishment and infection. Previous studies have focused on characterizing such effects on the vector–pathogen interface for an individual pathogen and its mosquito vector. In this study, we evaluated associations between immune responses to salivary proteins from non-dengue and non-malaria vector mosquito species with clinical characteristics of malaria and dengue, respectively. Surprisingly, antibody responses against Anopheles antigens in dengue patients correlated with red blood cell count and hematocrit, while antibody responses against *Aedes* proteins were associated with platelet count in malaria patients. Our data indicate that concurrent exposure to multiple disease-carrying mosquito vectors and their salivary proteins with differing immunomodulatory properties could influence the transmission, pathogenesis, and clinical presentation of malaria, dengue fever, and other vector-borne illnesses.

## 1. Introduction

Malaria and dengue fever are vector-borne diseases of significant public health concern in tropical and subtropical areas around the globe [1]. Dengue virus (DENV) is endemic in Central and South America, with occasional outbreaks in the US, particularly in Texas and Florida [2,3]. Importantly, several recent reports have indicated the occurrence of local malaria transmission in the continental US, while this disease remains endemic in Latin America [4,5]. 

During transmission, mosquito-borne pathogens are usually deposited in the skin of the vertebrate host along with arthropod saliva during blood-feeding [6]. Compelling evidence suggests that mosquito saliva induces an important immunomodulatory effect on vertebrate responses at the bite site, eventually systemically enhancing pathogen transmission [7,8]. Furthermore, immunogenic salivary proteins induce antibody responses that vary depending on age, seasonality, and vector abundance [9,10]. Thus, immunoglobulin G (IgG) antibody responses to mosquito salivary proteins have been used as a proxy for exposure to mosquito bites and indirect markers for disease risk [11,12], with some studies suggesting potential sex-dependent responses to mosquito saliva [13], but such findings have been partially explained by physiological factors, host genetics, and gender-related social determinants, resulting in differences in exposure [14,15]. With the increase in global mosquito-borne infections, a better understanding of sex-dependent host responses may be critical to mitigating the negative consequences of vector–pathogen transfer and hormone-related antigen responses.

Currently, control of vector-borne diseases relies heavily on decreasing human–vector contact through physical devices (i.e., bed nets) or insecticide treatment [16,17], since effective vaccines or drugs are scarce [18,19]. Importantly, the increase in insecticide and drug resistance calls for the design and implementation of new tools for disease control and new tools to estimate the potential risk of acquisition of vector-borne diseases, which will guide public health policy. Recently, an *Ae. aegypti* peptide, Nterm-34kDa, has been recommended as a tool to measure exposure to *Aedes* bites since a positive correlation was observed between the intensity of exposure, mosquito abundance, and anti-Nterm-34kDa IgG antibody levels [20,21]. Also, the gSG6-P1 peptide, identified from the *An. gambiae* SG6 salivary protein [22] has been extensively validated as a biomarker of exposure against *Anopheles* mosquitoes from the subgenus *Cellia* and *Anopheles* [23]. Both peptides have been used successfully to determine the level of exposure to mosquito bites associated with mosquito control interventions or risk of disease [12,24,25,26]. However, the SG6 protein is absent in the subgenus *Nyssorhynchus,* which includes *An. albimanus* and An. darlingi, we have designed several peptides from proteins present in these mosquitoes. Specifically, we have tested the *An. albimanus* peptides Peroxi-P1, Trans-1, and Trans-2 and the *An. darlingi* peptide AnDarApy-1, to be used in addition to the gSG6-P1 peptide, to have a better understanding of human–vector contact in Latin America. The peptides have previously been tested in several regions of Colombia, where we found that high IgG antibody levels are associated with malaria infection [27,28,29].

Prior work suggests that saliva induces skin responses associated with the potential for pathogen establishment [30]. In certain areas endemic for malaria or dengue, people can be exposed to hundreds of mosquito bites per day, and an increase in mosquito abundance is often associated with an increase in transmission [31]. It is well known that, in a specific geographical area, several mosquito species are in circulation simultaneously, although their feeding behavior may be different [32,33]. For instance, *Anopheles* mosquitoes are preferential nocturnal feeders, while *Aedes* mosquitoes are diurnal biters [34]. Therefore, it is possible that an individual may sustain *Aedes* mosquito bites during the day, followed by *Anopheles* mosquito bites at night with the potential for saliva from different mosquito species to have different immunomodulatory effects on the course of the infections with these pathogens [34]. To our knowledge, there are currently no studies that describe the effect of exposure to bites from different mosquito species on skin immune responses and pathogen replication. Thus, there is a critical need to better understand how contact with salivary proteins from different mosquito species due to sequential exposure to diurnal and nocturnal biters may impact human arbovirus acquisition and anti-viral immune responses. 

In this study, our primary goal was to evaluate the levels of antibodies against salivary antigens of different arthropod vectors of human disease in people with either malaria or dengue fever to assess whether responses against vector saliva are associated with blood parameters leading to severe clinical presentation. Since it is rare that only a single species of mosquito is found in a specific area, and several arthropod-borne diseases are common in the tropics, we hypothesized that exposure to salivary proteins with different immunomodulatory properties would impact infection and progression to disease. Transmission of Plasmodium and DENV often co-occur in tropical areas, mainly because of the overlap in the ecological niches preferred by the main vectors of these infections [35,36]. We, therefore, leveraged our ongoing malaria and dengue surveillance study in Norte de Santander, Colombia, to evaluate exposure to *Anopheles* and *Aedes* mosquito saliva and compare these data with blood parameters at diagnosis [37]. In this area, all DENV serotypes have circulated over the years. In addition, malaria caused by *Plasmodium falciparum* and *Plasmodium vivax* has been reported, with *P. vivax* as the most prevalent. To our knowledge, this is the first study exploring a potential correlation between exposure to the saliva of non-vectors in the clinical presentation of disease reflected by blood parameters.

## 2. Materials and Methods 

### 2.1. Human Sample Collection and Diagnosis

All protocols involving human subjects were reviewed and approved by Universidad de Pamplona and by the IRB of Kansas State University (IRB#1206). Written informed consent was obtained from all subjects, and blood samples were collected from each subject living in two areas with different endemicity levels for malaria and DENV in the department of Norte de Santander, Colombia. For the dengue study, we included samples from Los Patios (n = 76), Ocana (n = 19) and Cucuta (n = 29). For the malaria study, we included samples collected in Tibu (n = 34), Villa del Rosario (n = 3), and Tarra (n = 8). The sample size and ages of volunteers included in these studies are described in Table 1. Malaria diagnosis was completed using the Rapid Diagnostic Test Malaria (Xerion) and a thick blood smear evaluated by at least two experienced microscopists. Gametocyte carriage was performed by qRT-PCR on the Pvs25 and Pvs230 genes of *P. vivax* parasites, following the methods described elsewhere [38,39]. DENV diagnosis was performed using the Rapid Diagnostic Test, Cassette Dengue AG (Xerion) and confirmed by qRT-PCR [40]. We also tested 48 human samples collected from healthy volunteers living in Kansas (USA) and 55 living in Los Patios (Colombia) to measure antibody levels against the peptides in healthy/non-infected individuals living in both non-endemic areas (USA) and endemic areas (Colombia).

### 2.2. Salivary Antigens

Salivary gland extracts (SGE) from *An. albimanus* (STECLA strain) were prepared as previously published [41]. In this study, SGE and the previously reported salivary peptides Nterm-34kDa (*Ae. aegypti*) and gSG6-P1 (*An. gambiae*), AnDarApy-1 (*An. darlingi*) [28], Peroxi-P1, Trans1, and Trans-2 (*An. albimanus*) [27], were used to evaluate exposure to mosquito bites [27]. Peptides were synthesized by Genscript (Piscataway, NJ, USA), dissolved in ultrapure water, and frozen at −80 °C until used as antigens in ELISA assays.

### 2.3. Human IgG Antibody Detection by ELISA

The level of human IgG antibodies against mosquito salivary proteins was determined by an indirect ELISA, following the methods published by Londono-Renteria et al [29,41,42]. Briefly, 96-well ELISA plates (Nunc-MaxiSorp, Nalgene Nunc International, Rochester, NY, USA) were coated with 50 μL/well of *Ae. aegypti* or *An. albimanus* salivary gland extract (SGE) in a final concentration of 1 µg/mL prepared in a coating solution (1X PBS). Each salivary peptide was used in a final concentration of 2 µg/mL. Serum samples were tested in duplicate in a 1/100 dilution. After washes, plates were incubated with horseradish peroxidase-conjugated goat anti-human IgG (1: 1000) (Abcam, Ab81202), and colorimetric development was obtained using tetra-methyl-benzidine (one-solution micro-well, Gene-Script, Piscataway, NJ, USA). The reaction was terminated with 1 M phosphoric acid, and the absorbance was measured at 450 nm. Two controls were included on each plate: (1) control blank: two wells with antigen and without sample as a control for nonspecific induction of color for any of the reagents used in the test; and (2) positive control: 1 control per plate to test plate variation and normalize OD (optical density) values. IgG antibody levels are reported as ΔOD = Average patient OD value (duplicate) less the Blank OD.

### 2.4. Data Analysis

The median OD value was selected for all IgG levels against salivary antigens to determine high (above the median) or low (below the median) antibody levels. Differences between two independent groups were tested using the nonparametric Mann–Whitney *U* test. Correlation analysis between age and antigens was performed using the Spearman correlation test. All differences were considered significant at *p* < 0.05. All statistical tests were performed using Prism version 10 (Graph Pad Software Inc., La Jolla, CA, USA). 

## 3. Results

### 3.1. Plasmodium vivax Malaria and Exposure to the Non-Malaria Vector Aedes aegypti

**Figure 1:**
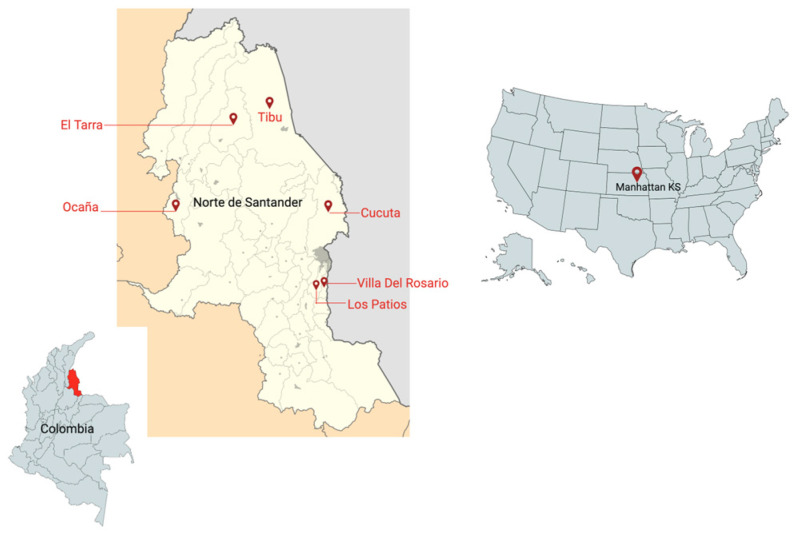
Map with the study sites in Colombia and the United States of America.

A total of 49 participants with current *P. vivax* malaria infections, diagnosed by microscopy and a rapid diagnostic test (RDT), from the areas of Tarra and Tibu in Norte de Santander, Colombia, were included in the study from 2018 to 2019 (Table 1) (Figure 1). 

The mean parasite count in our study population was 6665 parasites/µL (from 420 to 26,480 parasites/µL). Several malaria vectors circulate in our study area, including *An. albimanus* and *An. nunestovary* (*Nyssorhynchus*) and *An. pseudopunctipennis* and *An. punctimacula* (*Anopheles*), so we tested IgG antibodies using whole *An. albimanus* SGE, *An. albimanus*, *An. darlingi*, and gSG6-P1 peptides. We also measured exposure to *Aedes* bites in malaria-infected people, testing the IgG antibodies against the *Ae. aegypti* peptide Nterm34kDa. Since gametocytes are the Plasmodium parasite infective stages for the mosquito vector, we determined gametocyte carriage by measuring transcript levels of the Pvs25 and Pvs230 genes and comparing them with the IgG antibody levels against the mosquito salivary antigens. Our study did not reveal any correlation between the anti-saliva antibodies and parasite count or expression of the Pvs25 gene (gametes, ookinetes); however, IgG antibodies against *An. albimanus* SGE (Spearman correlation r_s_ = −0.6099, *p* = 0.0269), Trans-1 (r_s_ = −0.7510, *p* = 0.0031), gSG6-P1 (r_s_ = −0.6648, *p* = 0.0132), and Nterm34kDa (Spearman correlation r_s_ = −0.7253, *p* = 0.0050) showed a significant negative correlation with the level of expression of Pvs230 (gametocytes) in female participants (Table 2) (Supplementary Table S1). 

We next tested the correlation between IgG anti-salivary protein antibody levels and blood parameters such as red blood cell (RBC) count, white blood cell (WBC) count, platelet count, hemoglobin, hematocrit, and parasite count (Table 3) (Supplementary Table S2). We found that the levels of IgG antibodies against the *Ae. aegypti* peptide Nterm34kDa (non-malaria vector) were negatively correlated with RBC count in males, while females presented a significant positive correlation between anti-Nterm34kDa antibodies and WBC count. We also compared antibody levels with age and observed a positive correlation with the level of antibodies against whole *An. albimanus* SGE, gSG6-P1, and Trans-1 in females but not males (Table 4) (Supplementary Table S3). 

### 3.2. DENV and Exposure to the Non-DENV Vector Anopheles albimanus 

**Figure 2:**
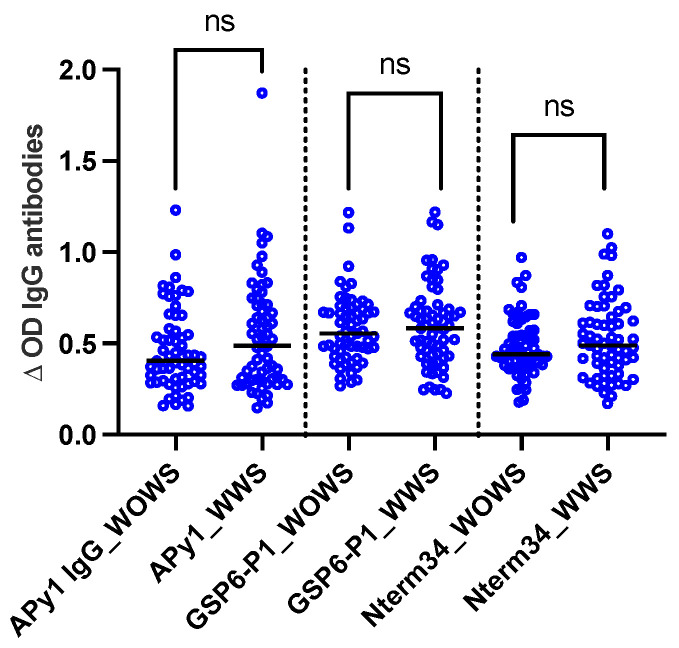
Scatterplot of IgG antibodies against the mosquito salivary peptides Nterm34kDa, AnDarApy1, and gSG6-P1 in people with dengue fever with warning signs (WWS) and dengue without warning signs. *Mann–Whitney test*, *p* > 0.05, ns = not significant.

A total of 124 DENV-positive volunteers living in Los Patios (n = 75), Cucuta (n = 24), and Ocana (n = 25) between October 2018 and September 2020 were included in this study. Following DENV classification according to WHO guidelines, we included 63 DENV patients with warning signs and 61 DENV patients without warning signs. We did not observe significant differences in the level of IgG antibody levels against Nterm34kDa, AnDarApy1, and gSG6-P1 peptides when comparing DENV groups (*Mann–Whitney test*, *p* > 0.05) (Figure 2). 

However, we observed a significant positive correlation between RBC count and IgG antibody levels against the Nterm34kDa peptide (Spearman correlation r_s_ = −0.2107, *p* = 0.0193) and gSG6-P1 (r_s_ = 0.1807, *p* = 0.0455) in males but not females. All comparisons are found in Table 5. 

### 3.3. Healthy Individuals from Endemic and Non-Endemic Areas 

**Figure 3:**
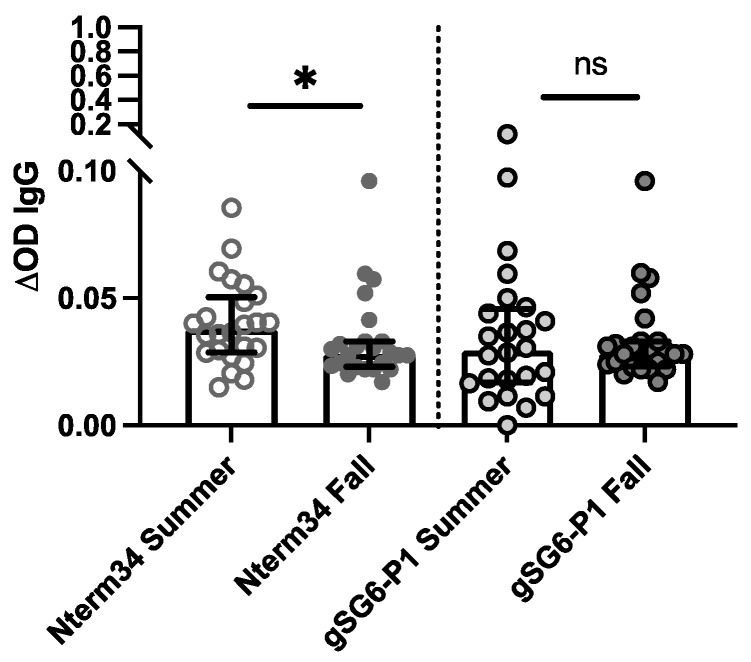
IgG antibody levels against the mosquito salivary peptides in healthy participants residents of Kansas (USA) in the summer of 2018 compared to levels in the IgG antibody levels found in volunteers followed during the Fall of 2018 (n = 25). Significance was measured by the Mann–Whitney test *p* < 0.05 (* = 0.0, and ns = not significant).

In 2018, we recruited participants in Manhattan, Kansas for a study focused on evaluating exposure to blood-sucking arthropods. A total of 27 participants donated blood samples in the summer (June–August) and fall (September–October). Our analyses revealed a significant reduction in antibody levels against Nterm-34kDa but not for gSG6-P1 in the Fall (Figure 3), suggesting an association between the intensity of exposure to mosquito bites and IgG antibody levels against mosquito salivary peptides. 

Next, we compared antibody levels in healthy US participants with levels in healthy individuals living in areas with endemic DENV and malaria. In 2018, samples were collected from 54 healthy volunteers living in houses where a DENV case was reported in Los Patios, Norte de Santander (Colombia). The samples were tested for the presence of asymptomatic infections through rapid tests and RT-PCR [43]. We observed a significant negative correlation between age and IgG antibodies against Nterm34kDa (r_s_ = 0.4182, *p* = 0.000) and gSG6-P1 (r_s_ = −0.3553, *p* = 0.0003). This significant negative correlation between age and anti-Nterm34kDa remained even when stratifying the data by gender (Table 5). When stratifying data by location, we observed that people from the US demonstrated significant negative correlations between antibody levels against both peptides and age, while healthy individuals from Colombia showed a positive correlation between age and gSG6-P1, but not Nterm-34kDa (Table 6).

### 3.4. Blood Parameters and Concordant Vector–Pathogen Interactions

**Figure 4:**
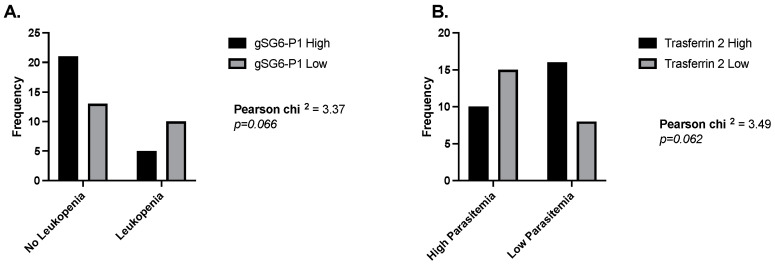
Odds ratios comparing levels of IgG antibodies against gSG6-P1 and Trans-2 peptide in people with and without leukemia (**A**) and low and high parasitemia levels (**B**).

We have described the blood parameters that highlight a potential correlation or association between exposure to a pathogen and its non-vector salivary antigens. However, we also observed interesting results when analyzing the IgG responses to the salivary antigens of *Anopheles* mosquitoes in malaria patients. Specifically, when measuring odds ratios, we found that malaria patients with normal leucocyte counts were 3.3 times more likely to present high IgG antibodies against the gSG6-P1 peptide (Figure 4A), and people with high parasitemia were 3.3 times more likely to have low antibodies against Trans-2 (Figure 4B). We also observed a significant negative correlation between platelet count and each *Anopheles* antigen in people with malaria. In the case of dengue patients and the *Aedes* peptide Nterm34kDa, we observed a significant positive correlation between this peptide and RBC count. 

## 4. Discussion

Mosquito saliva is composed of a plethora of molecules that are injected into the skin to counteract host responses and facilitate blood uptake. This saliva also induces the production of host antibodies that correlate with the intensity of mosquito exposure. Notably, levels of IgG antibodies against whole or specific salivary proteins have been categorized as a reliable tool to measure exposure to mosquito bites and disease transmission intensity [44,45]. In malaria, *Plasmodium* gametocytes in vertebrate blood are the infectious stages for the mosquito vector. Interestingly, previous studies showed that the carriage of specific parasite stages influenced host attractiveness to mosquitoes, with people who harbored gametocytes in their blood being more attractive to mosquitoes than those presenting parasitemia with only asexual stages [38,39,46,47]. Here, we evaluated gametocyte carriage by measuring the expression of *Plasmodium vivax* Pvs25, while Pvs230 was used to determine gametocyte carriage [48]. Upon analysis of the level of antibodies against mosquito saliva, we observed that the expression of Pvs230 was significantly negatively correlated with IgG antibody levels of all mosquito antigens tested in this study, including the non-malaria vector *Aedes* peptide Nterm34kDa. However, this association was not observed with Pvs25, suggesting that expression of Pvs230 may be associated with exposure to mosquito bites from both *Anopheles* and *Aedes* mosquitoes. Detection of parasite carriage in the malaria elimination era is crucial to determine the risk of reemergence or roadblocks in control interventions. Previous studies have determined gametocyte carriage by detecting transcripts of both Pvs25 and Pvs230. Although Pvs25 is expressed in the mosquito stages, there is evidence that the transcription of this gene starts at the mature gametocyte stage, but the protein is expressed only in the zygote when it is transforming into ookinete [48,49,50]. Studies suggest that Pvs25 is expressed in female gametocytes and Pvs230 is considered specific to male gametocytes [51]. Thus, we aimed to evaluate if there was a relationship between gametocyte carriage with these two genes. It was interesting to see a correlation between anti-saliva antibodies and Pvs230 but not Pvs25, and it may be associated with the fact that each male gametocyte (microgametocyte) maturates into eight microgametes while one female gametocyte (macrogametocyte) develops into one macrogamete [52]. However, more studies are needed to establish this correlation. 

Previous entomological data from our studies sites in Norte de Santander indicate that *Anopheles* and *Aedes* mosquitoes are prevalent in the area [53,54]. However, a limitation of this study is that the gSG6-P1 and the Nterm34kDa peptides we evaluated as markers of mosquito exposure have not been extensively validated for their correlation with mosquito abundance [55,56,57], and we did not collect specific mosquito data from the sites in our study, which could be used to confirm exposure to these mosquito species. 

One of the objectives of this study was to determine if antibody levels against mosquito saliva were correlated with blood parameters. In the case of the malaria cohort, we observed that IgG antibodies against the non-malaria vector *Aedes* peptide Nterm34kDa were negatively associated with RBC count. This finding is notable as one of the signs of severe malaria is anemia, yet we did not observe correlations between any of the *Anopheles* salivary antigens and RBC count. The observed negative correlation between IgG anti-Nter34kDa and RBC count suggests that individuals with the lowest RBC counts may have the highest exposure to non-malaria vector *Ae. aegypti* or may produce more antibodies against their saliva. Interestingly, a previous study reported that anemia accelerates blood intake by *Ae. aegypti*, although it may negatively impact egg production [58]. Also, prior work has demonstrated that DENV acquisition by *Ae. aegypti* was inversely correlated with iron concentration in human serum [59]. Thus, it is possible that DENV benefits from the *Aedes*’ preference to feed on anemic individuals. Indeed, among our DENV-infected participants, we observed a significant positive correlation between RBC and IgG antibody responses to both Nterm34kDa and gSG6-P1 peptides, suggesting increased exposure to mosquito bites while infected. Moreover, a recent study suggested that infection with DENV increased attractiveness for *Aedes* mosquitoes [60], similar to observations made with *Anopheles* mosquitoes and *Plasmodium* gametocyte carriers [39,46].

Although the *Ae. aegypti* peptide Nterm34kDa has been reported useful for evaluating exposure to *Aedes* mosquitoes around the world, the gSG6-P1 is restricted to the evaluation of exposure to the bite of *Anopheles* mosquitoes from the subgenera *Cellia* and *Anopheles* since the gSG6 protein is absent in the *Nyssorhynchus* subgenus. Thus, we designed several peptides to evaluate exposure to *An. darlingi* and *An. albimanus*, major members of this subgenus and important malaria vectors in Latin America [27,28]. However, we acknowledge that there is a potential for cross-reactivity when using these peptides. Specifically, we reported that the *An. darlingi* apyrase, where the peptides were designed, has a 63% identity with an apyrase of *An. gambiae* (AGAP011971), 49% with an apyrase from *Ae. aegypti* (AAEL006347), and an apyrase of *Cx. quinquefasciatus* (CPIJ011010), but no significant similarity was found with *An. albimanus* apyrases. However, the *An. darlingi* salivary oxidase/peroxidase has an 87.8% identity with the *An. albimanus* peroxidase (AAD22196.1), a 53% similarity with *An. gambiae* (AGAP010735), while the similarity with *Ae. aegypti* (AAEL000507) and *Cx. quinquefasciatus* (CPIJ017579) was 48% [27,28]. Thus, it is possible that some of the IgG antibodies measured against these *Anopheles*-based peptides may reflect some exposure to *Ae. aegypti*. Our group is currently evaluating the potential cross-reactivity of both *An. darlingi* and *An. albimanus* peptides with *Culex* and *Aedes* species based on entomological collections in Norte de Santander in hopes of validating the usefulness of such peptides in vector-borne endemic areas with multiple mosquito species. 

Mosquito abundance is associated with temperature, humidity, and other environmental factors [61,62,63]. In temperate and subtropical regions, mosquito abundance drastically changes in the summer vs. fall seasons, with higher mosquito populations in the former [64,65]. Previously, we observed a significant decrease in anti-tick antibodies during the fall months [66]. Interestingly, we found a significant decrease in IgG antibody levels against the Nter34kDa peptide in the fall compared to summer in people living in Kansas. Our results are in agreement with previous studies, which suggest a significant decrease in antibodies against *Ae. aegypti* mosquitoes after cessation of exposure [42]. However, we did not observe a decrease in antibodies against gSG6P1, although prior reports have indicated that antibodies against the gSG6 protein are short-lived^16^, suggesting a potential continued exposure. In Kansas, *An. quadrimaculatus* and *An. pseudopunctipennis* are abundantly found in the summer [67]. *Ae. aegypti* and *Ae. albopictus* have been collected in Kansas. However, only *Ae. albopictus* is found in Manhattan. Previous reports suggest that Nterm34kDa may cross-react with several *Aedes* species [21,40]. We were unable to find recent seasonal data on *Anopheles* captures, although a prior report describes the mosquito species present in Kansas from 1936 and 1937 [68]. Importantly, this previous report notes that extreme weather conditions were present in Kansas during the mosquito collections. Thus, it is possible that the species reported were behaving differently due to these unusual weather conditions. Despite the extreme weather conditions, the study reported several *Anopheles* species captured from June to November, which overlaps nicely with the two time points of our study (Summer 2018 (May–June) and Fall 2018 (September–November)) and could explain why the levels of antibodies against the gSG6-P1 peptide did not decrease during our follow-up. Because we could not find recent studies with the specific seasonality of *Anopheles* and *Aedes* mosquitoes endemic in Kansas, we also searched for any studies conducted in other countries with temperate climates in this hemisphere. A study in Northern Argentina indicated that *An. pseudopunctipennis* presents two peaks of abundance, one in the spring and another in fall/autumn [69]. Together, these studies provide a potential explanation for the persistence of antibody levels in samples collected in the early fall. Although this prior report did not include *Ae. aegypti* or *Ae. albopictus*, there is a description of *Ae. vexans* and *Ae. triseriatus* from the early summer to late fall, which could explain the minimal difference in antibody levels against the Nterm34kDa observed in our study. 

We observed that healthy volunteers living in a non-endemic area (Kansas) for either malaria or dengue (Kansas) presented negative correlations between age and anti-saliva antibodies, while healthy people living in Los Patios, Norte de Santander showed a positive correlation. We found a negative correlation between age and antibodies against *Ae. aegypti* whole salivary gland extract in healthy individuals living in Norte de Santander in 2015 [9] that could be associated with the development of tolerance as observed in other studies [45,70]. In this study, we included samples from different regions in the State of Norte de Santander. However, in another study we did not observe any associations with age [43], as observed in similar studies [13], suggesting that several factors may contribute to these discrepancies. Similarly, our study demonstrated that two different localities showed differences in antibody responses with age. Taking all these studies into consideration, it is possible that the age correlation is associated with local environmental and entomological data. Further larger studies need to be performed to establish the relationship between age and response to arthropod salivary antigens.

In addition to previous studies showing sex-associated differences in DENV incidence, geographical area also appears to strongly influence the rates of association and occurrence. For instance, a study including DENV cases from at least six Asian countries showed a higher incidence of dengue fever in males [71], while other studies in Central and South America demonstrated a higher incidence in females [72,73]. Herein, we observed that males and females respond differently to mosquito salivary antigens. These results may have implications for pathogen transmission and clinical disease presentation. A major implication of our study is that characteristics, including age, seasonality, and vector control must be considered when assessing serum levels of IgG against *Ae. aegypti* salivary proteins as a surrogate of the risk of human exposure to mosquito bites and pathogen transmission. Such information is needed in epidemiological studies aimed at control and prevention of mosquito-borne diseases. Furthermore, data on biological sex at the population level could potentially be used to inform calculations of the total disease burden in regions where vector-borne diseases have the highest impact.

We also observed an interesting association between blood parameters and antibody responses against the arthropod vector or both Plasmodium and DENV. First, we found a significant negative correlation between *Anopheles* antigens and platelets in malaria patients and a positive association between Nterm34kDa and RBC cells in dengue patients. This is interesting because, although platelets may be associated with pathology during malaria [74], usually RBC count and anemia are hallmarks of people living in malaria endemic areas. The opposite has been observed for dengue fever, where a low platelet count can be associated with a severe presentation of dengue fever [75,76]. Yet, previous studies have described a significantly lower platelet count in males with malaria during the high transmission season [77], suggesting a sex-dependent association. In general, thrombocytopenia in malaria has been associated with an increase in endothelial activation [78] and with a widespread activation of platelets by an interaction between the parasite PfEMP1 and the platelet’s CD36 and other receptors [79], but the sex-dependent relationships are still under investigation. Several anticoagulants in mosquito saliva are known to interact directly with platelets and other modulate endothelial responses [80,81], but the significance of the levels of antibodies against the *An. albimanus* antigens and not against gSG6-P1 or Nterm34kDa needs more investigation. In the case of IgG antibodies against Nterm34kDa and RBC, the association was positive in dengue patients but negative in malaria patients. One of the characteristics of malaria is the rupture of red blood cells during the parasite replication, leading to anemia, meaning a malaria patient already has characteristics in the blood that may be more attractive to the *Aedes* mosquitoes. Also, IgG antibodies against Nterm34kDa in a DENV-infected patient may have resulted from the infected bite—precluding the clinical presentation. The correlation observed in the malaria group does not mean that all *Aedes* mosquitoes will only bite anemic patients. Mosquitoes feed on the hosts that are available, and, in a malaria endemic area, there may be far more anemic individuals available, as compared to a dengue endemic area, where *Aedes* mosquitos will have to feed on the individuals that are available, whether they are anemic or not. Our primary objective in this study was, therefore, to start the conversation on how the presence of several species of mosquitoes in a determined area could affect the course of diseases carried by these species. Our observations lead us to believe that there are parameters that require more attention from the scientific community that may help us to make significant advances in the control of diseases like dengue and malaria.

In summary, our data indicate that individuals living in malaria and dengue endemic areas possess antibodies against the salivary proteins from several different mosquito species that reside within these areas. Moreover, exposure to malaria and dengue mosquito vectors may be associated with clinical and immune responses to infection with the reciprocal pathogens transmitted by these specific species. Thus, our findings suggest that concurrent exposure to multiple disease-carrying mosquito vectors and their salivary proteins with differing immunomodulatory properties could influence the transmission and pathogenesis of malaria, dengue fever, and other vector-borne illnesses. A better understanding of the molecular mechanisms underlying how exposure to multiple and sequential bites from numerous mosquito species influences immune responses and pathogen transmission will advance the development of immune-targeting interventions to reduce disease spread. Additionally, including data on antibody responses against the main mosquito species found in specific areas will provide new knowledge on the interplay of such species, pathogen transmission, and disease severity in humans, which will help to inform the development of more effective vector control and disease prevention efforts.

## 5. Conclusions

Antibodies against salivary proteins are reliable markers for exposure intensity to bites from insect vectors. The data we have presented here support the use of IgG antibodies against salivary proteins as biomarkers of the intensity of exposure to mosquito bites and further indicate that these measurements can provide critical knowledge on how exposure to mosquito salivary proteins influences the transmission of vector-borne diseases. Moreover, including the potential effects of sequential exposure to saliva from different mosquito species will promote a better understanding of the pathogenesis of diseases borne by the vectors themselves as well as in the context of simultaneous exposure to non-vector mosquitos. 

## Figures and Tables

**Table 1:** Description of the study population ages in the malaria endemic areas (Tibu and Tarra, Norte de Santander, Colombia) and dengue endemic area (Los Patios and Cucuta, Norte de Santander, Colombia) and healthy individuals from Los Patios (Colombia) and Manhattan, Kansas (USA).

Infection Status	All Age Years (Range)	Female (Range)	Male (Range)
Malaria	33.4 (1–67), n = 49	36.0 (16–53), n = 14	32.4 (1–67), n = 35
Dengue	15.3 (1–76), n = 124	17.6 (1–76), n = 70	12.4 (1–69), n = 54
Healthy	28.9 (2–79), n = 103	27.0 (2–79), n = 65	32.3 (2–72), n = 38

**Table 2:** Correlation analysis between IgG antibody responses against the *Ae. Aegypti* peptide Nterm34kDa and parasite count/gametocytemia by gender in Plasmodium-positive volunteers. Data are presented using Spearman correlation r_s_ and significance *p* < 0.005.

Nterm-34kDa	Pvs25	Pvs230	Parasite Count
All	−0.0109 (*p* = 0.9473)	−0.1553 (*p* = 0.3453)	−0.0314 (*p* = 0.8495)
Females	−0.5000 (*p* = 0.0819)	−0.7253 (*p* = 0.0050)	−0.3077 (*p* = 0.3064)
Males	0.1829 (*p* = 0.3711)	0.0715 (*p* = 0.7287)	0.2151 (*p* = 0.2913)

**Table 3:** Correlation analysis between IgG antibody responses against *Ae. aegypti* Nterm34kDa peptide and blood parameters by gender in *Plasmodium*-positive volunteers. Data are presented using Spearman correlation r_s_ and significance *p* < 0.005.

Nterm-34kDa	Red Blood Cell Count	White Blood Cell Count	Platelet Count	Hemoglobin	Hematocrit
**All**	−0.3288 (*p* = 0.0313)	0.2363 (*p* = 0.1271)	−0.2078 (*p* = 0.1812)	−0.1402 (*p* = 0.3699)	−0.1374 (*p* = 0.3797)
**Females**	−01560 (*p* = 0.5942)	0.5923 (*p* = 0.0256)	−0.5560 (*p* = 0.0389)	−0.0683 (*p* = 0.8166)	−0.0989 (*p* = 0.7366)
**Males**	−0.4171 (*p* = 0.0244)	0.0561 (*p* = 0.7727)	−0.0017 (*p* = 0.9929)	−0.1594 (*p* = 0.4090)	−0.1264 (*p* = 0.5135)

**Table 4:** Correlation analysis between IgG antibody responses against each peptide and age by gender in *P. vivax*-infected patients. Data are presented using Spearman correlation r_s_ and significance *p* < 0.005.

Peptide	Age
**All**	
Peroxi-P1	−0.0207 (*p* = 0.8880)
Trans-1	0.3270 (*p* = 0.0218)
Trans-2	0.1596 (*p* = 2733)
An. albimanus SGE	0.0471 (*p* = 0.07478)
gSG6-P1	0.2064 (*p* = 0.1547)
Nterm-34kDa	0.0828 (*p* = 0.5719)
**Females**	
Peroxi-P1	0.3645 (*p* = 0.2001)
Trans-1	0.6196 (*p* = 0.0181)
Trans-2	0.3934 (*p* = 0.1641)
*An. albimanus* SGE	0.5934 (*p* = 0.0253)
gSG6-P1	0.5334 (*p* = 0.0495)
Nterm-34kDa	0.2356 (*p* = 0.4175)
**Males**	
Peroxi-P1	−0.1172 (*p* = 0.5027)
Trans-1	0.2603 (*p* = 0.1310)
Trans-2	0.0793 (*p* = 0.6507)
An. albimanus SGE	−0.0771 (*p* = 0.6599)
gSG6-P1	0.1113 (*p* = 0.5243)
Nterm-34kDa	0.0417 (*p* = 0.8121)

**Table 5:** Correlation analysis between IgG antibody responses against *Anopheles* antigens and blood parameters by gender. In dengue fever patients. Data are presented using Spearman correlation r_s_ and significance *p* < 0.005.

Peptide	Red Blood Cell Count	White Blood Cell Count	Platelet Count	Hemoglobin	Hematocrit
**All**					
**AnDarApy-1**	0.0955 (*p* = 0.2934)	0.0277 (*p* = 0.7607)	−0.0831 (*p* = 0.3609)	0.0999 (*p* = 0.2715)	0.1092 (*p* = 0.2294)
**gSG6-P1**	0.1807 (*p* = 0.0455)	−0.0829 (*p* = 0.3617)	−0.0484 (*p* = 0.5949)	0.0921 (*p* = 0.3112)	−0.1097 (*p* = 0.2269)
**Females**					
**AnDarApy-1**	−0.0571 (*p* = 0.6390)	−0.0641 (*p* = 0.5980)	0.0739 (*p* = 0.5434)	0.0847 (*p* = 0.4858)	0.0520 (*p* = 0. 6689)
**gSG6-P1**	0.0216 (*p* = 0.8591)	−0.1233 (*p* = 0.3092)	0.0338 (*p* = 0.7815)	0.0015 (*p* = 0.9899)	−0.0156 (*p* = 0.8981)
**Males**					
**AnDarApy-1**	0.2797 (*p* = 0.0425)	0.2063 (*p* = 0.1382)	−0.3052 (*p* = 0.0263)	0.1138 (*p* = 0.4173)	0.1681 (*p* = 0.2290)
**gSG6-P1**	0.4099 (*p* = 0.0023)	0.0559 (*p* = 0.6908)	−0.2038 (*p* = 0.1433)	0.2074 (*p* = 0.1362)	0.2408 (*p* = 0.0824)

**Table 6:** Correlation analysis between IgG antibody responses against each peptide and age. Data are presented using Spearman correlation r_s_.

Peptide	Age
**All**	
**gSG6-P1**	−0.3533 (*p* = 0.0003)
**Nterm-34kDa**	−0.4182 (*p* = 0.0000)
**Correlations by gender**	
**Females**	
**gSG6-P1**	−0.3575 (*p* = 0.0035)
**Nterm-34kDa**	−0.4087 (*p* = 0.0007)
**Males**	
**gSG6-P1**	−0.3702 (*p* = 0.0370)
**Nterm-34kDa**	−0.4105 (*p* = 0.0196)
**Correlations by location**	
**Colombia**	
**gSG6-P1**	0.2702 (*p* = 0.0481)
**Nterm-34kDa**	0.0796 (*p* = 0.5675)
**US**	
**gSG6-P1**	−0.4772 (*p* = 0.0005)
**Nterm-34kDa**	−0.4049 (*p* = 0.0039)

## Data Availability

The data presented in this study is available as a Supplementary data.

## Supplementary Materials

The following supporting information can be downloaded at: https://www.mdpi.com/article/10.3390/pathogens13010052/s1, Table S1: Correlation analysis between IgG antibody responses against each *Anopheles* salivary antigen and parasite count/gametocytemia by gender in Plasmodium positive volunteers. Data is presented in Spearman r_s_ and significance *p* < 0.005; Table S2: Correlation analysis between IgG antibody responses against Anopheles peptides and blood parameters by gender in *Plasmodium* positive volunteers. Data is presented in Spearman correlation r_s_ and significance *p* < 0.005; Table S3: Correlation analysis between IgG antibody responses against the Ae. aegypti peptide Nterm34kDa and blood parameters by gender. In dengue fever patients. Data is presented in Spearman correlation r_s_ and significance *p* < 0.005; Supplementary data S4.
