# Supplementary material for: Mosquito Salivary Antigens and Their Relationship to Dengue and P. vivax Malaria
Source: Pathogens. 2024 Jan 5;13(1):52. doi: 10.3390/pathogens13010052 (PMC10818852; doi:10.3390/pathogens13010052)
Supplement: Supplementary file 1 [file pathogens-13-00052-s001.zip › Supplementary data/Supplementary Table S1.pdf]

Supplementary Table S1: Correlation analysis between IgG antibody responses against each *Anopheles* salivary antigen and parasite count/gametocytemia by gender in Plasmodium positive volunteers. Data is presented in Spearman ϱ and significance *p<0.005*.

Peptide	Pvs25	Pvs230	Parasite count
**All**			
**Peroxi-P1**	0.0121 (p=0.9415)	-0.2842 (p=0.0795)	-0.0989 (p=0.5492)
**Trans-1**	0.0715 (p=0.6655)	-0.1355 (p=0.4107)	-0.1987 (p=0.2254)
**Trans-2**	-0.1937 (p=0.2373)	-0.0644 (p=0.6970)	-0.0984 (p=0.5511)
**An. albimanus SGE**	-0.0497 (p=0.7638)	-0.3072 (p=0.0571)	-0.0538 (p=0.7451)
**gSG6-P1**	-0.0901 (p=0.5855)	-0.2913 (p=0.0720)	-0.1123 (p=0.4960)
**Females**			
**Peroxi-P1**	-0.0824 (p=0.7890)	-0.5440 (p=0.0546)	-0.2418 (p=0.4262)
**Trans-1**	-0.3989 (p=0.1770)	-0.7510 (p=0.0031)	-0.1761 (p=0.5650)
**Trans-2**	0.1978 (p=0.5171)	-0.3187 (p=0.2886)	-0.0165 (p=0.9574)
**An. albimanus SGE**	-0.2802 (p=0.3538)	-0.6099 (p=0.0269)	-0.1593 (p=0.6031)
**gSG6-P1**	-0.3132 (p=0.2974)	-0.6648 (p=0.0132)	-0.1593 (p=0.6031)
**Males**			
**Peroxi-P1**	0.0510 (p=0.8047)	-0.1772 (p=0.3866)	0.0395 (p=0.8480)
**Trans-1**	0.2113 (p=0.3001)	0.1217 (p=0.5356)	-0.1498 (p=0.4651)
**Trans-2**	0.2205 (p=0.2790)	0.1022 (p=0.6193)	-0.0728 (p=0.7236)
**An. albimanus SGE**	0.0168 (p=0.9353)	-0.1733 (p=0.3971)	-0.0147 (p=0.9432)
**gSG6-P1**	-0.0544 (p=0.7919)	-0.1433 (p=0.4850)	-0.0332 (p=0.8722)
