# Supplementary material for: Mosquito Salivary Antigens and Their Relationship to Dengue and P. vivax Malaria
Source: Pathogens. 2024 Jan 5;13(1):52. doi: 10.3390/pathogens13010052 (PMC10818852; doi:10.3390/pathogens13010052)
Supplement: Supplementary file 1 [file pathogens-13-00052-s001.zip › Supplementary data/Supplementary Table S2.pdf]

Supplementary Table S2: Correlation analysis between IgG antibody responses against Anopheles peptides and blood parameters by gender in *Plasmodium* positive volunteers. Data is presented in Spearman correlation ϱ and significance *p<0.005*.

Peptide	Red blood cell count	White blood cell count	Platelet count	Haemoglobin	Haematocrit
**All**					
**Peroxi-P1**	-0.2660 (p=0.0847)	-0.0269 (p=0.8642)	-0.3167 (p=0.0385)	-0.1691 (p=0.2782)	-0.2145 (p=0.1671)
**Trans-1**	-0.2462 (p=0.1115)	0.2950 (p=0.0548)	-0.3288 (p=0.0314)	-0.1691 (p=0.2782)	-0.2445 (p=0.1141)
**Trans-2**	-0.2651 (p=0.0858)	0.1180 (p=0.4510)	-0.3651 (p=0.0161)	-0.2651 (p=0.0974)	-0.3740 (p=0.0135)
**An. albimanus SGE**	-0.1076 (p=0.4923)	0.1589 (p=0.3088)	-0.311 (p=0.0420)	-0.1158 (p=0.4597)	-0.1609 (p=0.3028)
**gSG6-P1**	-2769 (p=0.0723)	0.3205 (p=0.0361)	-0.2636 (p=0.0877)	-0.1189 (p=0.4477)	-0.1300 (p=0.4061)
**Females**					
**Peroxi-P1**	-0.3714 (p=0.1910)	0.6497 (p=0.0119)	-0.4330 (p=1220)	-0.1454 (p=0.62000	-0.2571 (p=0. 3748)
**Trans-1**	-0.1958 (p=0.5023)	0.7522 (p=0.0019)	-0.5391 (p=0.0467)	0.1235 (p=0.6741)	-0.0726 (p=0.8052)
**Trans-2**	-0.3099 (p=0.2809)	0.6144 (p=0.0194)	-0.2308 (p=0.4273)	-0.1410 (p=0.6307)	-0.3099 (p=0.2809)
**An. albimanus SGE**	-0.0330 (p=0.9109)	0.6763 (p=0.0079)	-0.4857 (p=0.0783)	0.1828 (p=0.5316)	0.0857 (p=0.7708)
**gSG6-P1**	-0.0857 (p=0.7708)	0.6188 (p=0.0153)	-0.3626 (p=0.2026)	0.1564 (p=0.5834)	0.1121 (p=0.7028)
**Males**					
**Peroxi-P1**	-0.3224 (p=0.0880)	-0.2376 (p=0.2145)	-0.2266 (p=0.2371)	-0.2902 (p=0.1268)	-0.2572 (p=0.1780)
**Trans-1**	-0.2645 (p=0.1656)	0.1199 (p=0.5356)	-0.02303 (p=0.2294)	-0.2740 (p=0.1504)	-0.3069 (p=0.1053)
**Trans-2**	-0.2334 (p=0.2230)	-0.0712 (p=0.7135)	-0.3548 (p=0.0589)	-0.2930 9p=0.1229)	-0.3972 (p=0.0329)
**An. albimanus SGE**	-0.1429 (p=0.4595)	0.0094 (p=0.9615)	-0.1947 (p=0.3116)	-0.2169 (p=0.2584)	-0.2341 (p=0.2216)
**gSG6-P1**	-0.3300 (p=0.0804)	0.2192 (p=0.2534)	-0.1062 (p=0.5834)	-0.1468 (p=0.4443)	-0.1417 (p=0.4634)
