# Supplementary material for: Mosquito Salivary Antigens and Their Relationship to Dengue and P. vivax Malaria
Source: Pathogens. 2024 Jan 5;13(1):52. doi: 10.3390/pathogens13010052 (PMC10818852; doi:10.3390/pathogens13010052)
Supplement: Supplementary file 1 [file pathogens-13-00052-s001.zip › Supplementary data/Supplementary Table S3.pdf]

Supplementary Table S3: Correlation analysis between IgG antibody responses against the *Ae. aegypti* peptide Nterm34kDA and blood parameters by gender. In dengue fever patients. Data is presented in Spearman correlation ϱ

Peptide	Red blood cell count	White blood cell count	Platelet count	Haemoglobin	Haematocrit
**All**					
**Nterm-34kDa**	0.2107 (p=0.0193)	-0.0429 (p=0.6375)	-0.0649 (p=0.4754)	0.1543 (p=0.0885)	0.1587 (p=0.0797)
**Females**					
**Nterm-34kDa**	0.0741 (p=0.5422)	0.0480 (p=0.6932)	-0.0153 (p=0.9001)	0.1431 (p=0.2373)	0.01078 (p=0.3474)
**Males**					
**Nterm-34kDa**	0.3904 (p=0.0039)	0.0304 (p=0.8289)	-0.1611 (p=0.2493)	0.1922 (p=0.1680)	0.2288 (p=0.0994)
